# Correction: A single-molecule counting approach for convenient and ultrasensitive measurement of restriction digest efficiencies

**DOI:** 10.1371/journal.pone.0308550

**Published:** 2024-08-05

**Authors:** Yi Zhang, Takuro Nunoura, Daisuke Nishiura, Miho Hirai, Shigeru Shimamura, Kanako Kurosawa, Chieko Ishiwata, Shigeru Deguchi

In the Template DNA preparation subsection of Materials and methods, there is an error in the first and second sentences of the second paragraph. The correct sentences are: The upstream and downstream three nucleobases flanking BmtI site of mNeonGreen template were modified from original ATG (i.e., ATGGCTAGCATG) to TTT (i.e., TTTGCTAGCTTT) or GGG (i.e., GGGGCTAGCGGG), where the underlined region is BmtI recognition site, using QuikChange lightning site-directed mutagenesis kit (Agilent). The primer set for the TTT mutagenesis was TTGCTGTCCACCAGTAAAGCTAGCAAAACCATGATGATGATGATGATGAGAACC and GGTTCTCATCATCATCATCATCATGGTTTTGCTAGCTTTACTGGTGGACAGCAA, and the primer set for the GGG mutagenesis was CCCATTTGCTGTCCACCAGTCCCGCTAGCCCCACCATGATGATGATGATGATG and CATCATCATCATCATCATGGTGGGGCTAGCGGGACTGGTGGACAGCAAATGGG, where the underlined bases are the mutations.

In the DNA cleavage by CRISPR-Cas9 subsection of Materials and methods, there is an error in the second sentence of the paragraph. The correct sentence is: For a comparison purpose, two forward primers (CCTCTAATACGACTCACTATAGGCGATGACGATAAGGATCCGAGTTTAAGAGCTATGC; CCT CCTCTAATACGACTCACTATAGGTCGCCTTTCCAGGCCGCCAGTTTAAGAGCTATGC) were designed to prepare the DNA template used for the in vitro transcription of the corresponding guide RNA (sgRNA) targeting the respective sites (underlined sequences of the above primers) of mNeonGreen DNA template.
