# Supplementary material for: A single-molecule counting approach for convenient and ultrasensitive measurement of restriction digest efficiencies
Source: PLoS One. 2020 Dec 31;15(12):e0244464. doi: 10.1371/journal.pone.0244464 (PMC7775078; doi:10.1371/journal.pone.0244464)
Supplement: S1 Appendix — (PDF) [file pone.0244464.s001.pdf]

**S1 Appendix. A theoretical number of template DNA molecules remaining undigested.**

The initial concentration of template DNA: $c = 1\ \mu g/50\mu L = 20\ ng/\mu L$;

The molecular weight of mNeonGreen template DNA (http://biotools.nubic.northwestern.edu/OligoCalc.html): $MW = 622740\ g/mol$;

When the digest efficiency is $r = 95\%$;

The number of template DNA molecules remaining undigested per 1 μL is: $n_0 = (1 - r) \cdot \frac{c}{MW} \cdot N_A \cdot 1\ \mu L = (1 - 0.95) \times \frac{20\times10^{-9}}{622740} \times 6.02 \times 10^{23} \times 1 = 9.7 \times 10^8 \approx 10^9$.
