# Supplementary material for: A single-molecule counting approach for convenient and ultrasensitive measurement of restriction digest efficiencies
Source: PLoS One. 2020 Dec 31;15(12):e0244464. doi: 10.1371/journal.pone.0244464 (PMC7775078; doi:10.1371/journal.pone.0244464)
Supplement: S2 Appendix — (PDF) [file pone.0244464.s002.pdf]

S2 Appendix. A theoretical number of false positive clones in molecular cloning experiments.

We can envision a general transformation experiment using a plasmid DNA (e.g., 3 kb) carrying an antibiotic resistance gene. An undigested plasmid may result in a false positive. Typical transformation efficiency of 1×10^7 cfu/μg is roughly equivalent to 1 out of every $(\frac{10^{-6}\,g}{3000 \times 660\,g/mol} \times 6.02 \times 10^{23}\,mol^{-1})/(1 \times 10^7) \approx 30000$ molecules of the plasmid used being transformed. If we add 1 μL of the digested DNA solution (containing 92 pM undigested plasmid DNA) into an aliquot of competent cells before plating, $\frac{92 \times 10^{-12}\,mol/L \times 10^{-6}\,L \times 6.02 \times 10^{23}\,mol^{-1}}{30000} \approx 1800$ undigested plasmid DNA molecules may be taken up by the competent cells.
