# Supplementary material for: A single-molecule counting approach for convenient and ultrasensitive measurement of restriction digest efficiencies
Source: PLoS One. 2020 Dec 31;15(12):e0244464. doi: 10.1371/journal.pone.0244464 (PMC7775078; doi:10.1371/journal.pone.0244464)
Supplement: S3 Appendix — (PDF) [file pone.0244464.s003.pdf]

S3 Appendix. $P_{positive}$ calculation.

The molar concentration of 20 ng/μL DNA solution: $c' = c/MW = 3.21 \times 10^4\ pM$;

Initial molar concentration after 1000-fold dilution (100-fold dilution with H_2O followed by further 10-fold dilution with CFPS solution): $c_0 = c'/1000 = 32.1\ pM$;

Droplet volume: $v = \frac{\pi}{4} \cdot d^2 \cdot h = 37.7\ fL$, where $d = 4\ \mu m$ is the diameter of the microchamber, $h = 3\ \mu m$ is the depth of the microchamber;

The average number of DNA molecules per 1 droplet: $\lambda = c_0 \cdot v \cdot N_A = 32.1 \times 10^{-12} \times 37.7 \times 10^{-15} \times 6.02 \times 10^{23} = 0.7$, where N_A is the Avogadro constant;

Therefore, the theoretical proportion of positive droplets (i.e., the cumulative probability) is: $P(k \geq 1) = 1 - P(k = 0) = 1 - e^{-\lambda} = 0.50$.
