# Supplementary figures and images for: A single-molecule counting approach for convenient and ultrasensitive measurement of restriction digest efficiencies
Source: PLoS One. 2020 Dec 31;15(12):e0244464. doi: 10.1371/journal.pone.0244464 (PMC7775078; doi:10.1371/journal.pone.0244464)

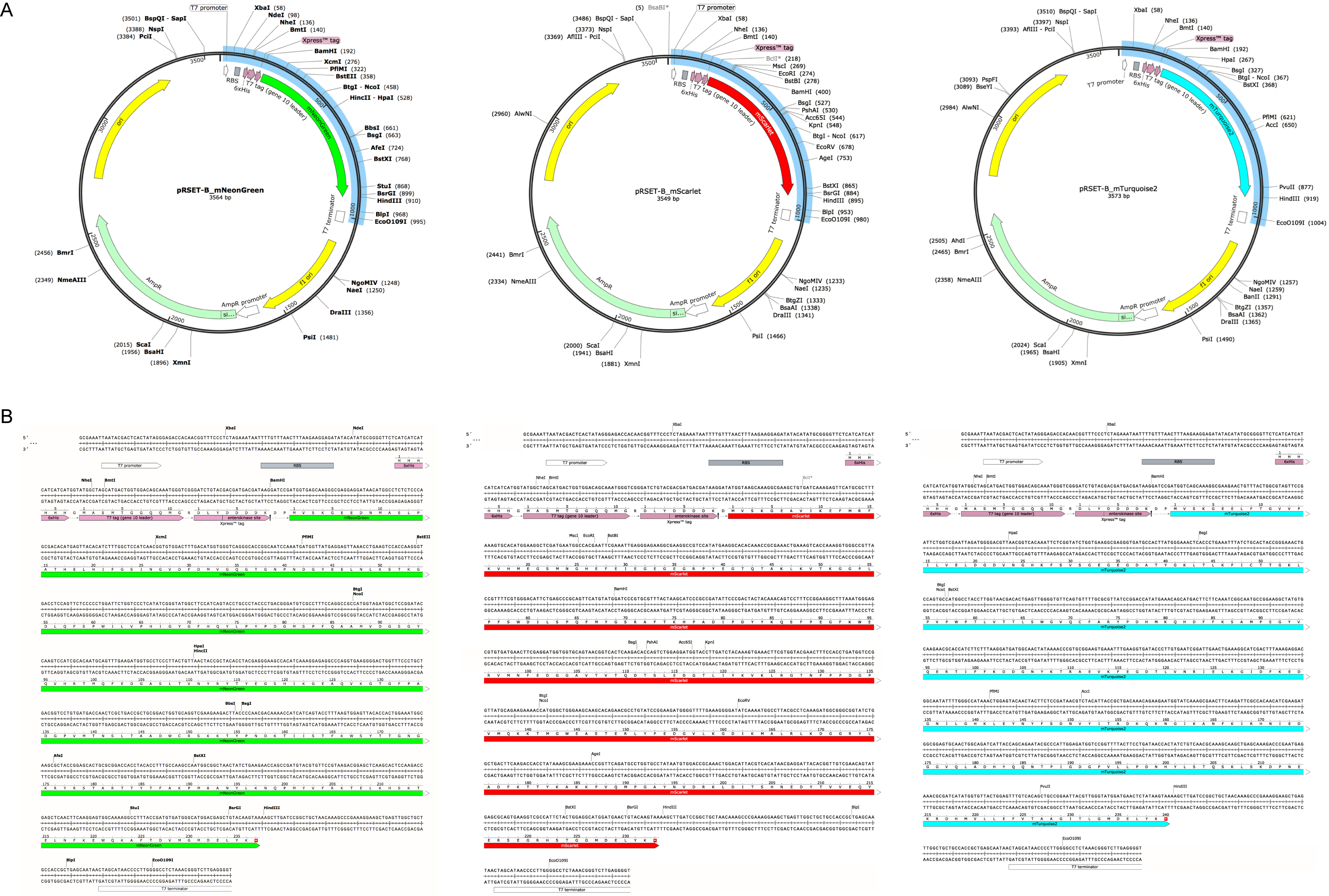

Supplement: S1 Fig — (A) An overview of the expression plasmid maps for mNeonGreen, mScarlet, and mTurquoise2. The translucent blue-shaded region is amplified using PCR to generate the linear DNA used for restriction digestion and CFPS reactions. (B) The sequences of the linear template DNA for mNeonGreen, mScarlet, and mTurquoise2. Unique 6+ cutters are marked on the sequence. (TIF) [file pone.0244464.s004.tif]

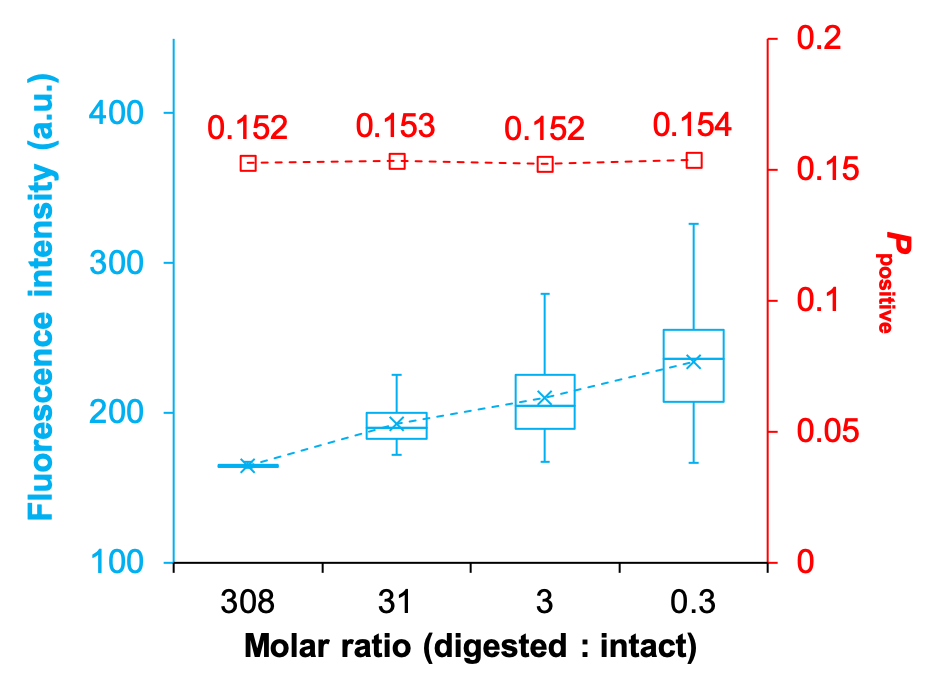

Supplement: S2 Fig — Full-length (1008 bp) mNeonGreen template DNA was prepared with PCR, purified from the PCR solution, and quantified by NanoDrop (133 ng/uL). It was diluted 1000 times in H2O to an arbitrary concentration of 214 pM, which is equivalent to 0.16 DNA molecules per droplet if adding 0.5 μL to a 15 μL CFPS mix (i.e., further diluted 30 times). In parallel, two fragments in a NcoI-digested DNA solution were separated by gel electrophoresis, purified from the gel for each, and quantified by NanoDrop. The relatively large fragment (~550 bp) and the relatively small fragment (~440 bp) had different mass concentrations but had similar molar concentrations (6.6×10^4^ pM). These two DNA fragments were serially diluted in H2O. Each of the (diluted or undiluted) fragments was added 0.5 μL to the CFPS mix, resulting in a molar ratio of full-length DNA: each DNA fragment from 214: 6.6×10^4^ to 214: 6.6×101, i.e., from 1: 308 to 3: 1. The experiment did not further increase the proportion of the digested DNA because the available concentration of gel-purified DNA is limited in practice. As shown in the box plot (blue colored), the absolute fluorescence intensity of individual positive droplets was reduced with the increase of the proportion of digested DNA, which is attributable to the resource consumption on the truncated peptide synthesis. The blue cross marker in the box is the mean of each group. In spite of the co-encapsulation of the interferential DNA (in this case, the small fragment containing T7P and RBS) in the droplet, the proportion of positive droplets remained unchanged (red colored) and was consistent with the input concentration of the full-length template DNA (theoretical Ppositive = 1−e−0.16≈0.15). All molecular weights were pre-calculated using an online tool http://biotools.nubic.northwestern.edu/OligoCalc.html. (TIF) [file pone.0244464.s005.tif]

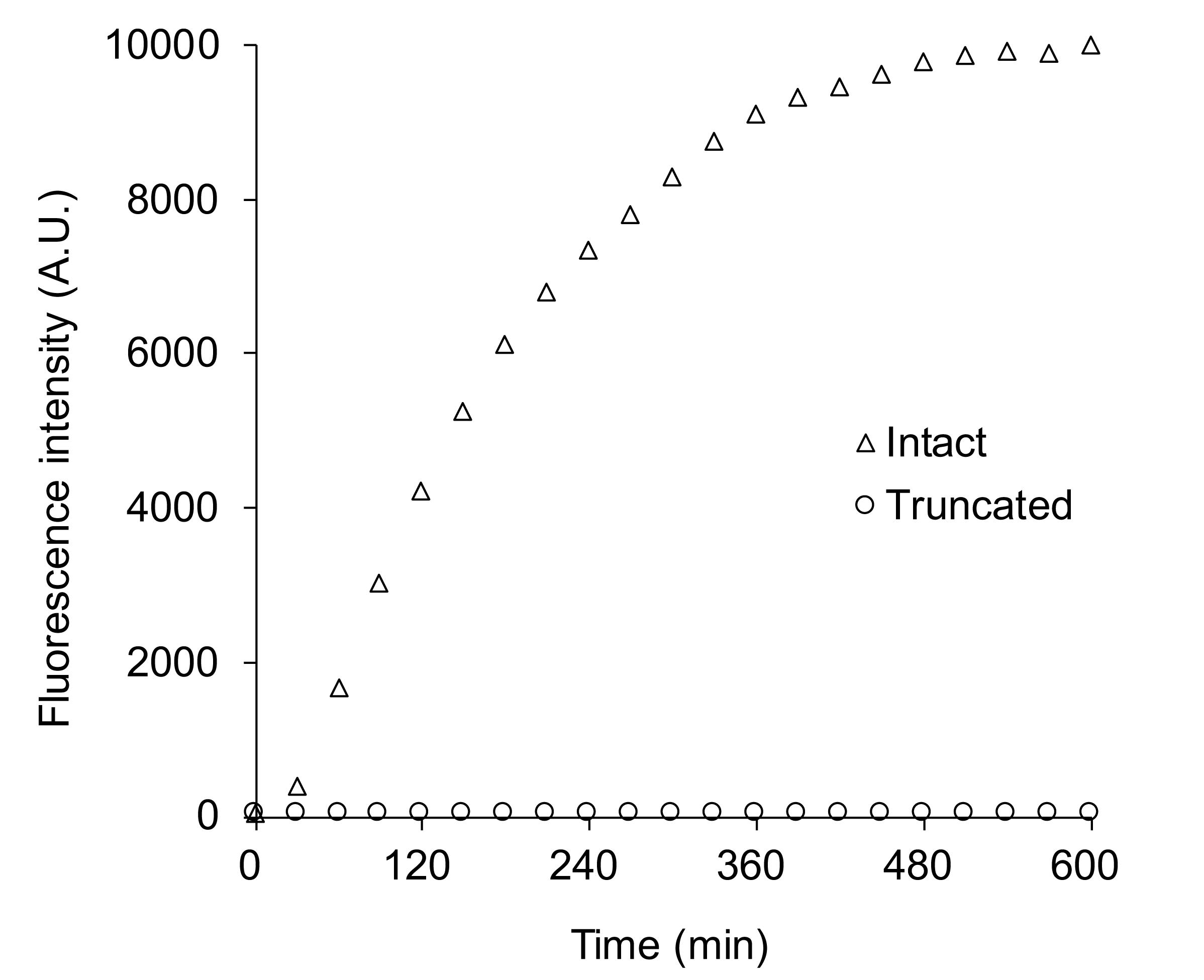

Supplement: S3 Fig — The mNeonGreen template was digested by NcoI-HF, and the N-terminus fragment was purified from the agarose gel. The intact and the truncated template DNA contain the same T7P, RBS, and the sequences located upstream of the start codon. An equal quantity (68 ng) of DNA was added into a 15 μL CFPS solution. The CFPS reaction was carried out in a microtiter plate (Non-binding, μClear, 384-well plate, Greiner Bio-One) at room temperature for a sufficiently long time (10 h). The fluorescence intensity was recorded using a microplate reader (Synergy, BioTek) with an excitation filter (485/20 nm) and an emission filter (528/20 nm). The run-off transcript from the NcoI-digested template was translated to a truncated polypeptide. In contrast to the intact mNeonGreen, the truncated one showed no fluorescence increase over time. (TIF) [file pone.0244464.s006.tif]

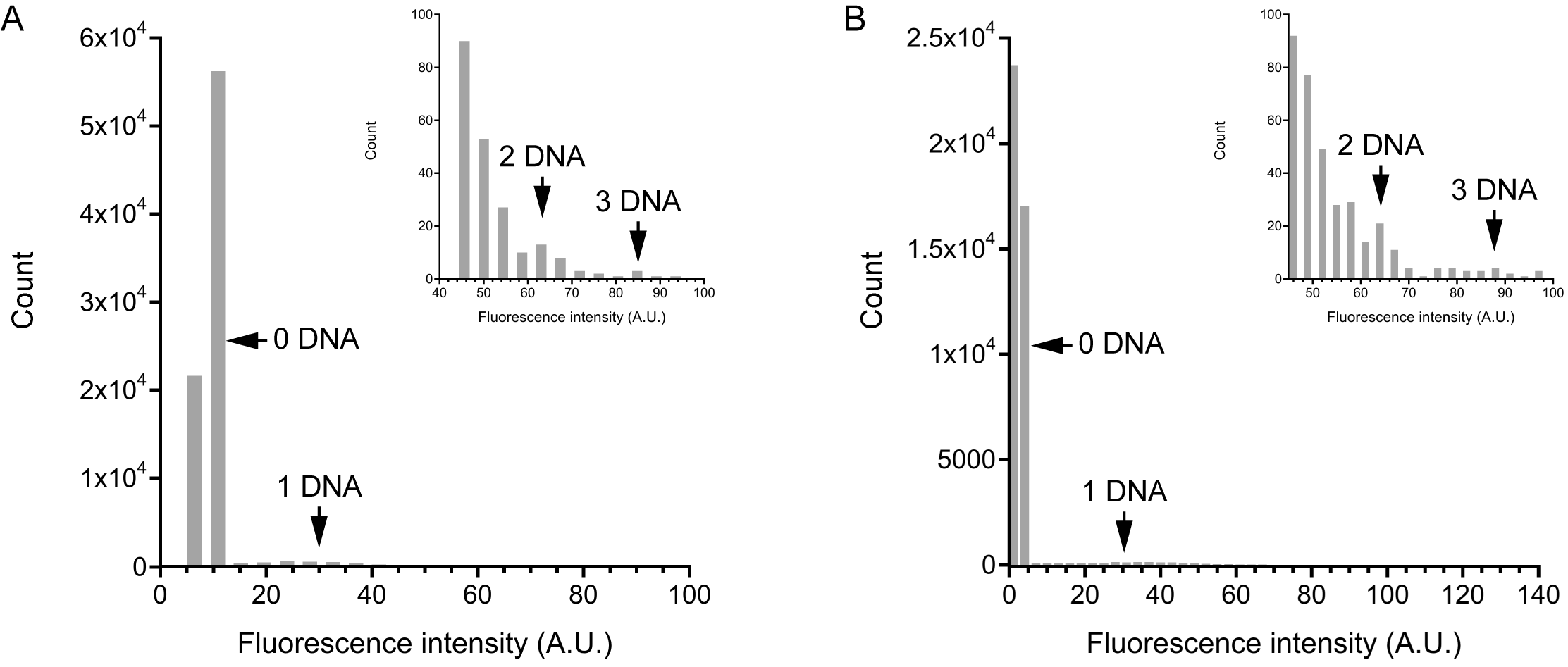

Supplement: S4 Fig — (A) Digital protein synthesis using NcoI-digested mNeonGreen DNA solution without heat-inactivation. (B) Digital protein synthesis using NcoI-digested mNeonGreen DNA solution with heat-inactivation (80 oC, 20 min). Discrete distribution of fluorescence intensity can be observed in both cases, as a result of the stochastic distribution of DNA molecules into the droplets. From the histogram, the fraction of positive droplets (containing undigested DNA molecules) in both cases was the same with each other (Ppositive [heat-inactivation] = 3112/81408 ≈ 0.038; Ppositive [heating-free inactivation] = 1533/40693 ≈ 0.038). These results suggested there is no remarkable difference between heat-inactivation and heating-free inactivation. (TIF) [file pone.0244464.s007.tif]

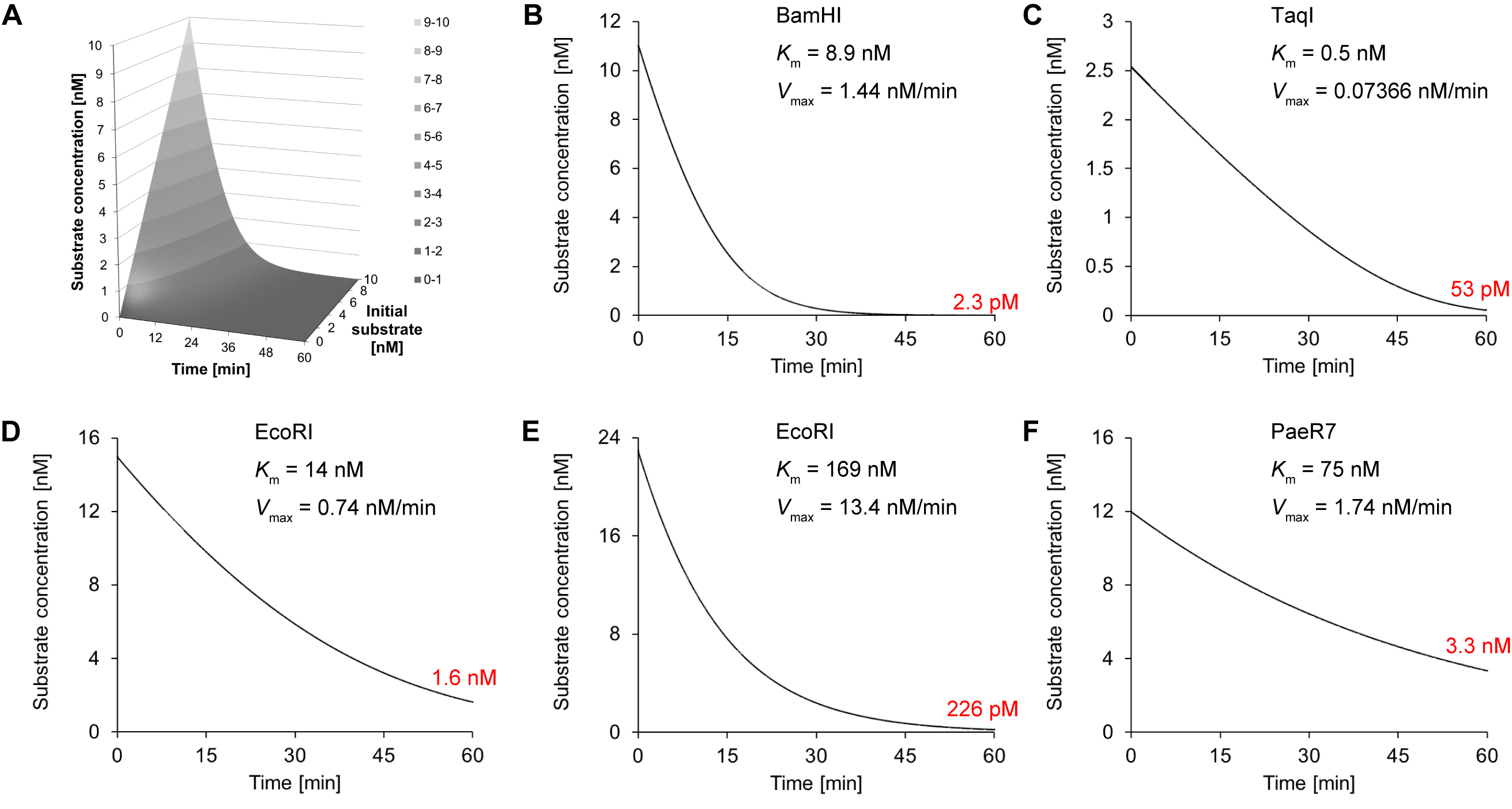

Supplement: S5 Fig — (A) The concentration of residual template DNA (vertical axis) associated with the digestion time (horizontal axis) and initial DNA concentration (depth axis). We applied the enzyme kinetic constants (Km and Vmax) that were determined by previous studies to the omega function of BamHI (B) [27], TaqI (C) [24], EcoRI (D, E) [26, 28], and PaeR7 (F) [25], respectively. The kinetic constants for S5A Fig were arbitrarily from the report of BamHI. Only a tiny portion of restriction endonucleases’ kinetic constants have been measured in the past. The initial concentrations (as shown at the time-point zero of the horizontal axis) of template DNA were also taken from the respective reports. The determination of kinetic constants of restriction endonucleases is highly susceptible to the buffer components, reaction conditions, DNA sequences, and so on. As exemplified by the well-studied EcoRI (S5D Fig), our theoretical prediction was in good agreement with a previously reported real-time cleavage measurement [26], reflecting the reliability of the omega function. From this numerical calculation, we can speculate that there should be 3 pM ~ 3 nM DNA remaining undigested for a given DNA sample with an initial concentration of about 3 ~ 30 nM. This estimation was in good agreement with our digital counting results (see Table 1). (TIF) [file pone.0244464.s008.tif]

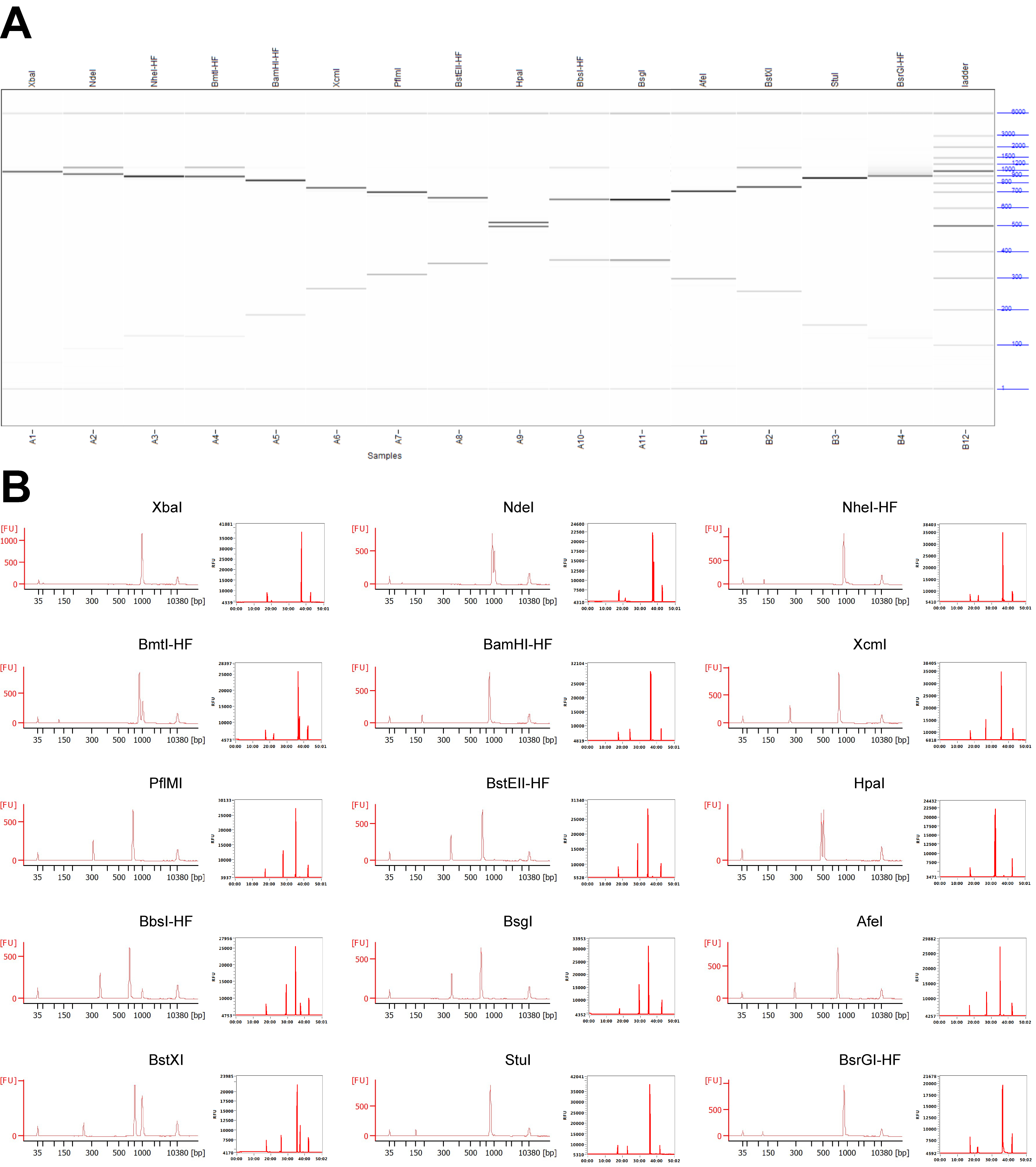

Supplement: S6 Fig — (A) Capillary electrophoresis by Fragment Analyzer (Advanced Analytical Technologies). The same sample was analysed by Bioanalyzer in parallel (see Fig 3 in the main text). The reconstructed gel image clearly showed that the result of Fragment Analyzer was essentially identical to the result of Bioanalyzer. (B) Electropherograms of each DNA sample. Each group is composed of two electropherograms. The left one is Bioanalzyer’s electropherogram, and the right one is Fragment Analyzer’s electropherogram. The peak number, the relative peak height, and the relative peak intervals all indicated the identical performance between Bioanalyzer and Fragment Analyzer for analyzing the restriction digests. (TIF) [file pone.0244464.s009.tif]

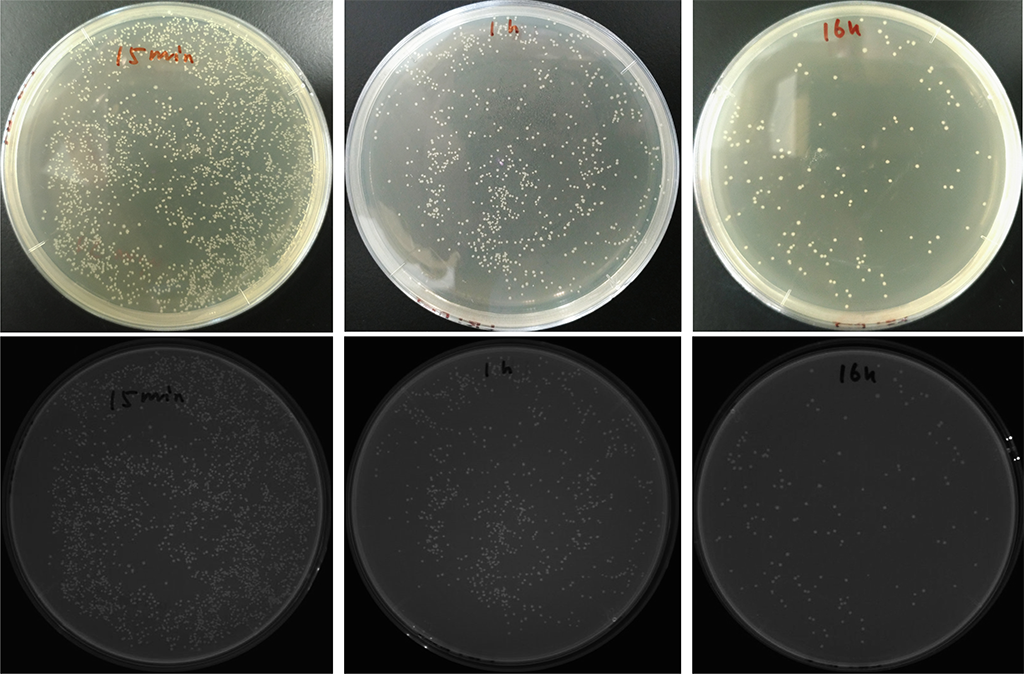

Supplement: S7 Fig — The mNeonGreen expression plasmid was digested by NdeI for 15 min (left), 1 h (middle), and 16 h (right), respectively. An equal quantity of each digest was added into an equal aliquot of competent cells and plated together onto an ampicillin-selective LB-agar plate. The upper three are bright-field images, and the lower three are the corresponding fluorescence images under the illumination of 365 nm ultraviolet light. (TIF) [file pone.0244464.s010.tif]

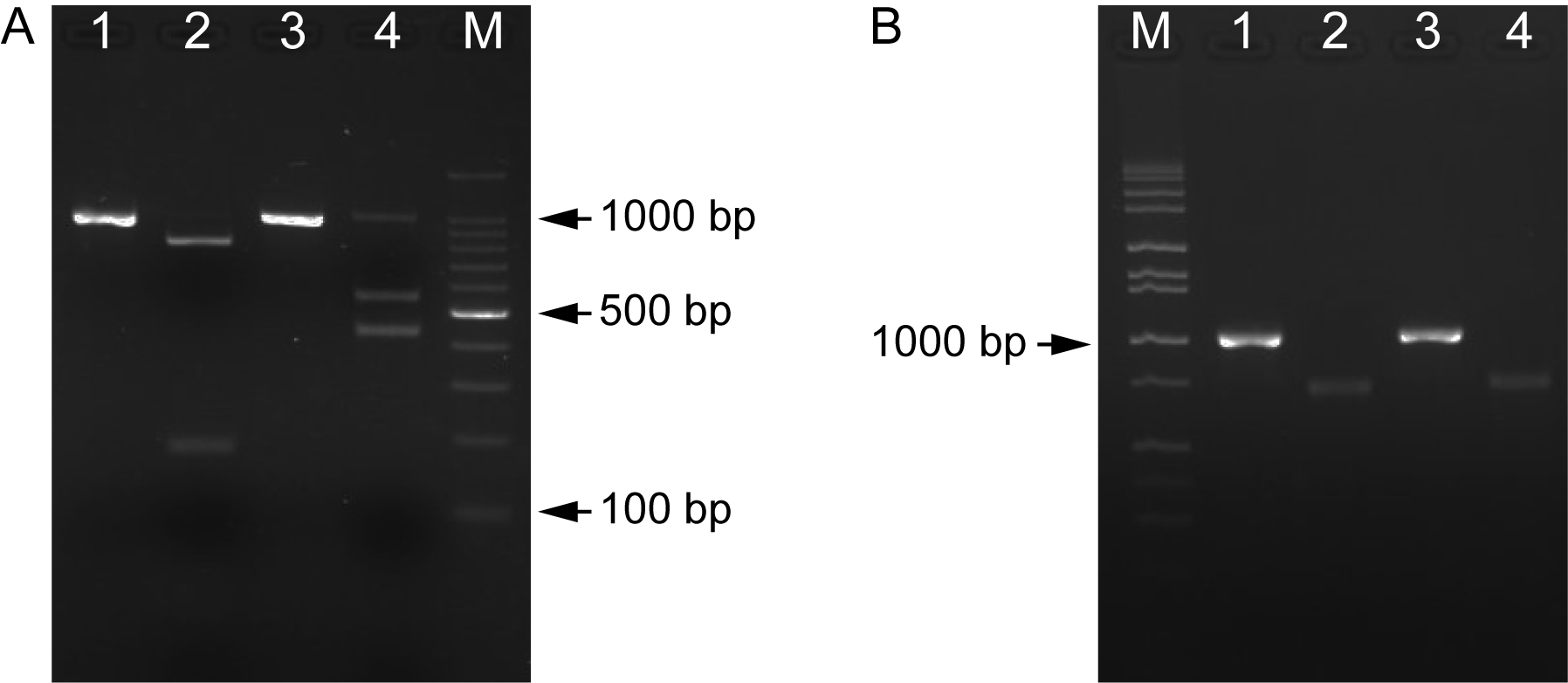

Supplement: S8 Fig — Agarose gel electrophoresis for DNA digests generated by CRISPR-Cas9 (A) or strandase (B). (A) Lane M: 100 bp DNA ladder; lane 1 & 3: linear mNeonGreen template DNA; lane 2: cleaved by Cas9-sgRNA targeting a site around BamHI, producing two DNA fragments about 180 bp and 820 bp; lane 4: cleaved by Cas9-sgRNA targeting another site around NcoI/BtgI, producing two DNA fragments about 440 bp and 560 bp. (B) Lane M: Hi-Lo DNA marker; lane 1: linear mNeonGreen template DNA phosphorylated at 5'-end of the antisense strand; lane 2: sense ssDNA; lane 3: linear mNeonGreen template DNA phosphorylated at 5'-end of the sense strand; lane 4: antisense ssDNA. In general, fluorogenic intercalators stains the ssDNA much less efficiently than for dsDNA. All lanes were loaded with 100 ng DNA sample. (TIF) [file pone.0244464.s011.tif]

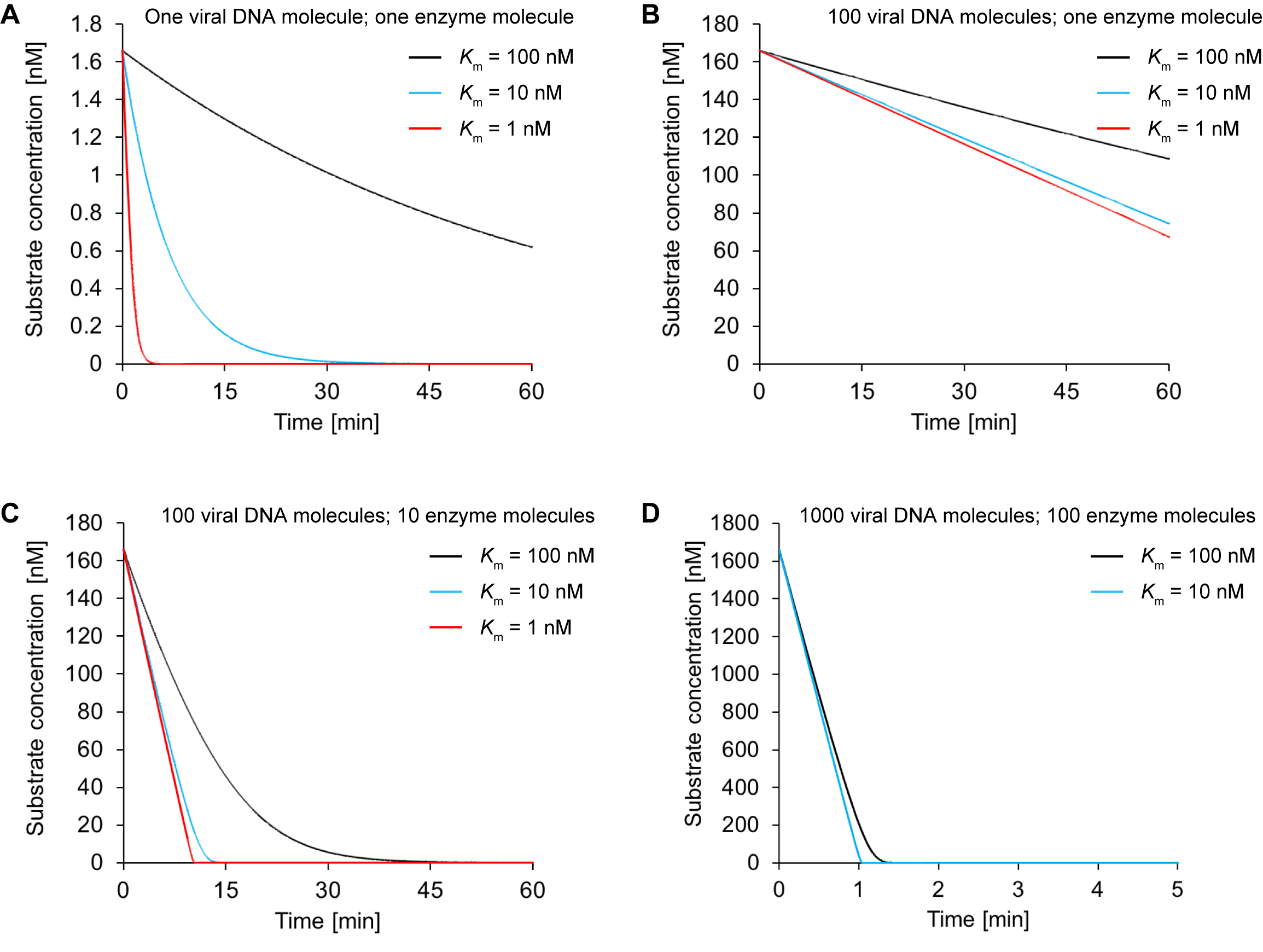

Supplement: S9 Fig — Differing from the instances listed in S5 Fig, the calculation here was based on an “putative” enzyme, instead of the experimentally measured ones. The turnover number of the putative enzyme was assumed to be 1 min-1. The viral DNA molecule was assumed to contain only one cleavage site. The amount of uncleaved DNA can be largely affected by the number of viral DNA molecules, the number of enzyme molecules, and the Michaelis constant (Km). We gave herein several representative situations as “one DNA molecule and one enzyme molecule (A)”, “many DNA molecule and a few enzyme molecules (B)”, “many DNA molecules and many enzyme molecules (C)”, and “much more DNA molecules and much more enzyme molecules (D)”. Because the calculation for the case of Km = 1 nM exceeded the calculation capacity of Microsoft Excel, we only showed the other two in S9D Fig. (TIF) [file pone.0244464.s012.tif]
