# Supplementary material for: A single-molecule counting approach for convenient and ultrasensitive measurement of restriction digest efficiencies
Source: PLoS One. 2020 Dec 31;15(12):e0244464. doi: 10.1371/journal.pone.0244464 (PMC7775078; doi:10.1371/journal.pone.0244464)
Supplement: S1 Raw images — (PDF) [file pone.0244464.s015.pdf]

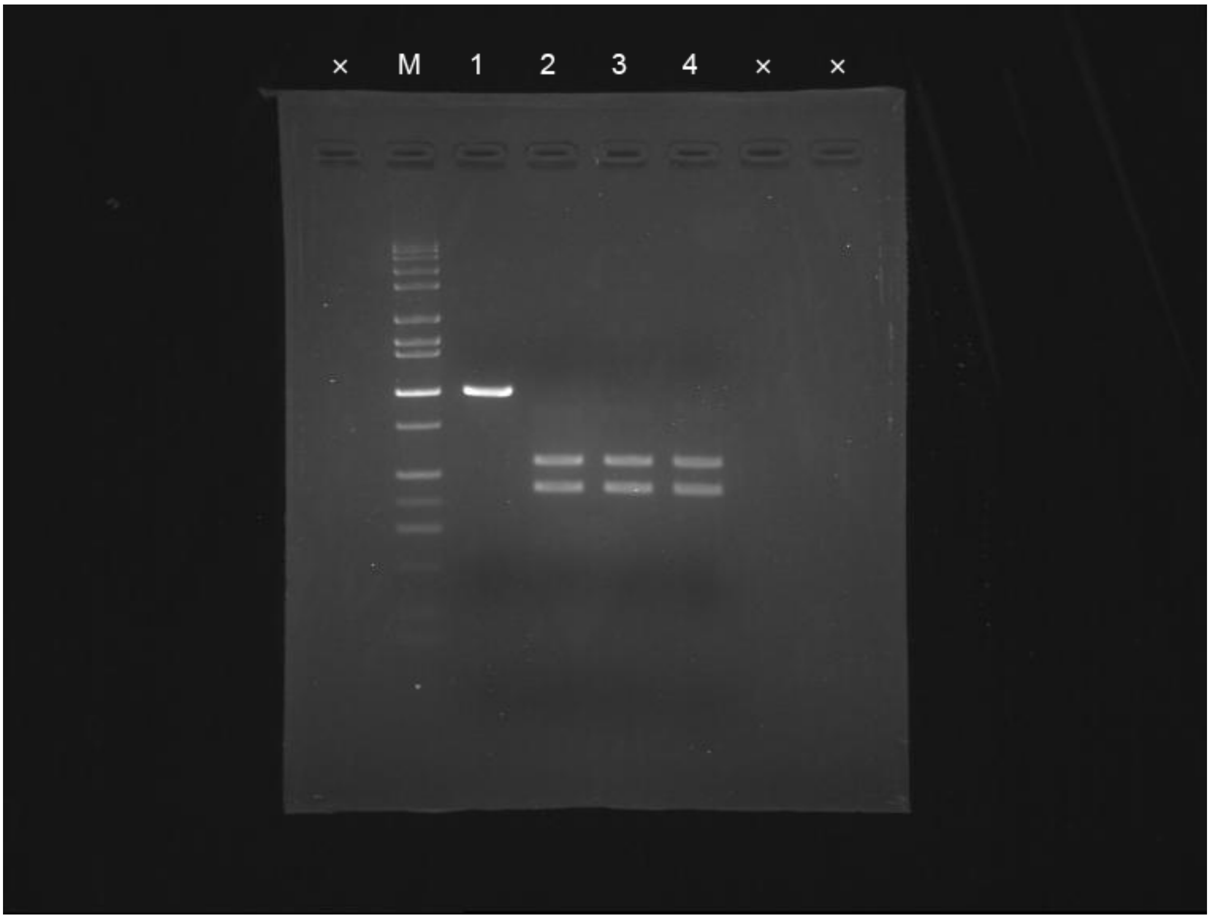

The above is the uncropped image for the left part of Fig 1A. The labels on each lane are the same as the ones in Fig 1A. Empty lanes marked with “×” were not included in the final figure.

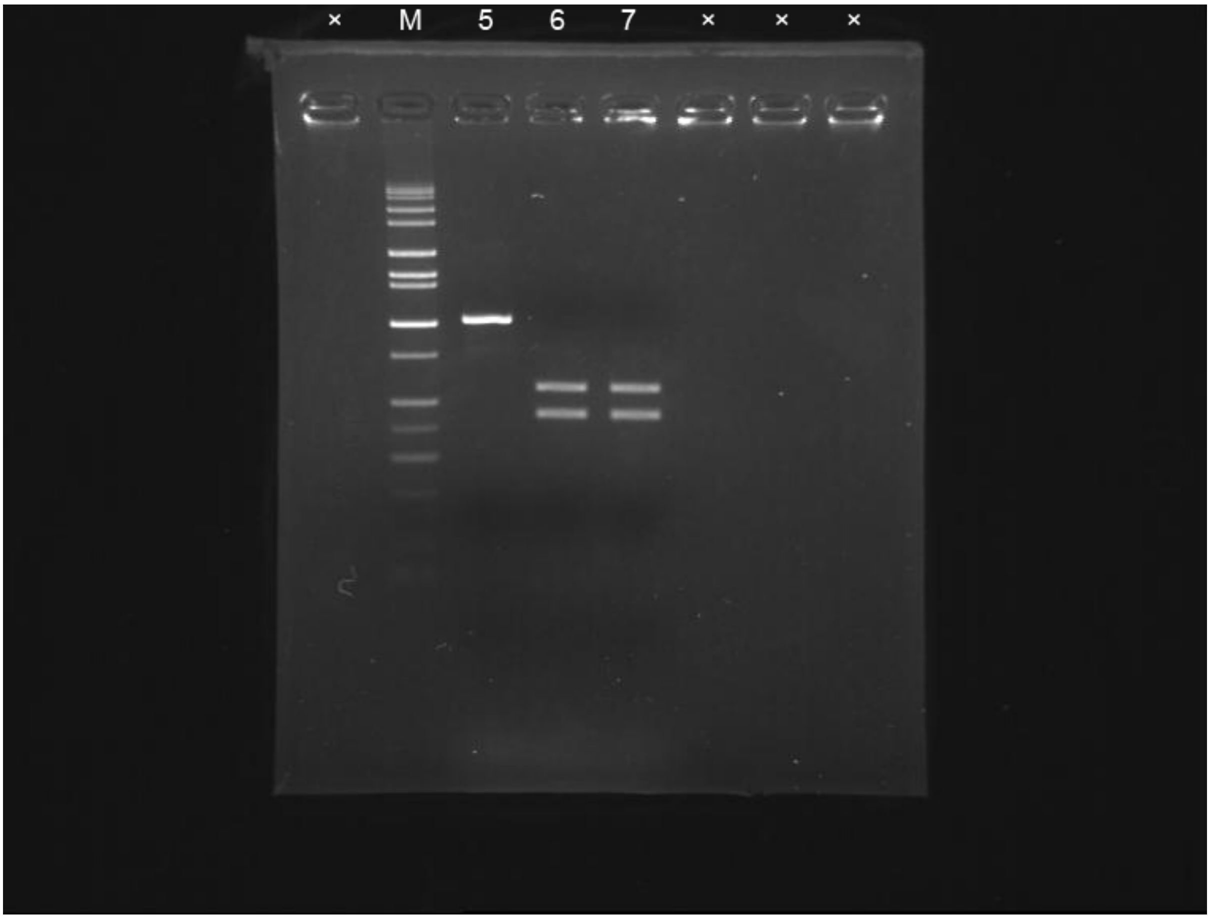

The above is the uncropped image for the right part of Fig 1A. The labels on each lane are the same as the ones in Fig 1A. Empty lanes marked with "×" were not included in the final figure.

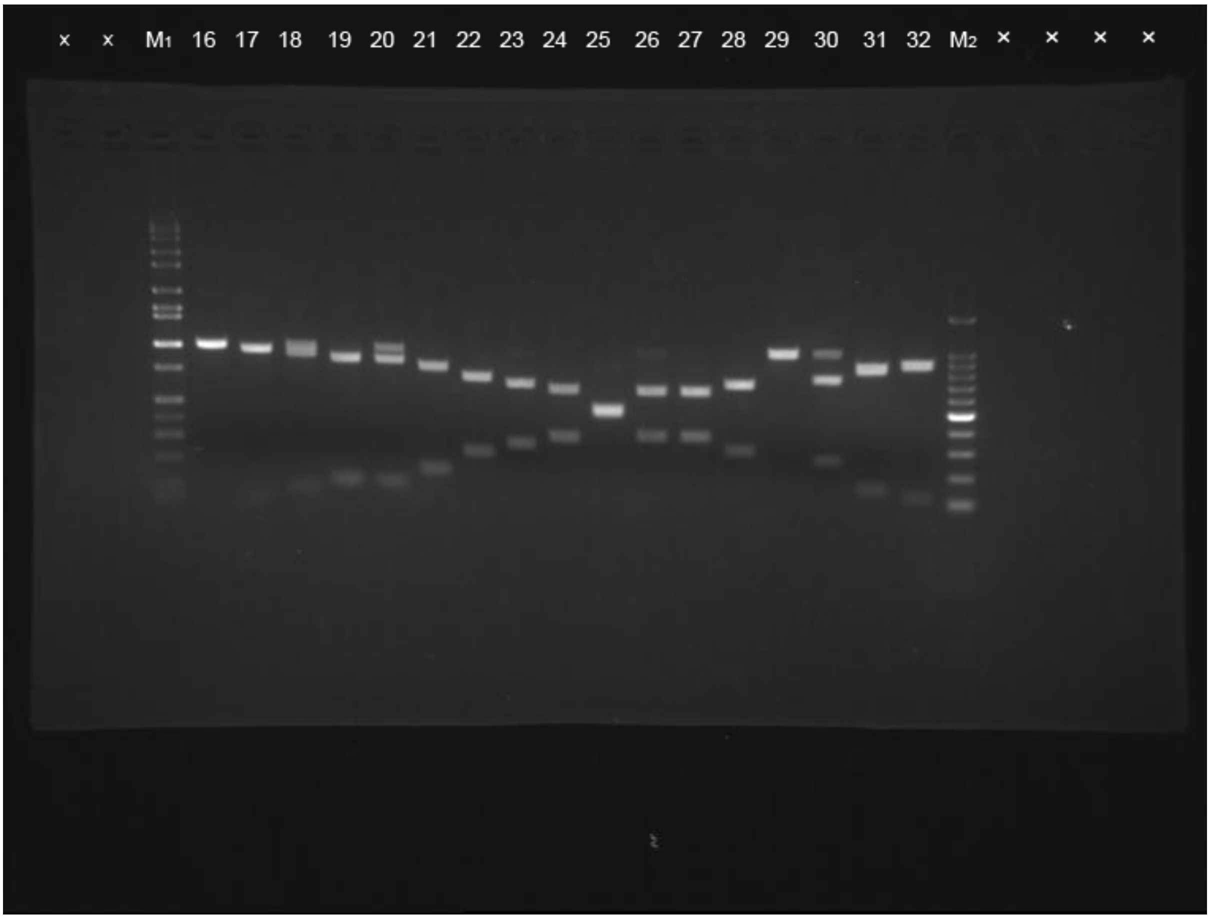

The above is the uncropped image for the bottom part of Fig 3. The labels on each lane are the same as the ones in Fig 3. Empty lanes marked with "×" were not included in the final figure.

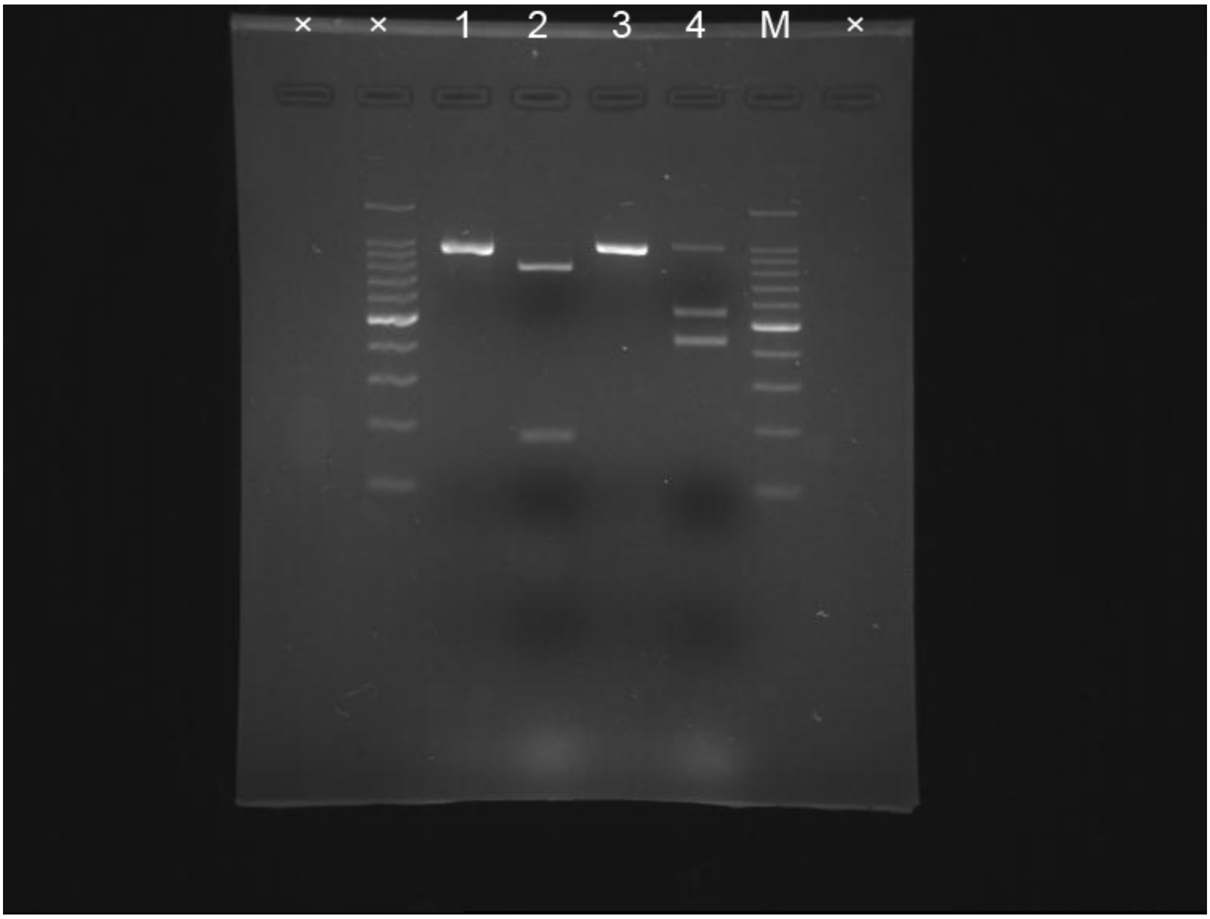

The above is the uncropped image for S8A Fig. The labels on each lane are the same as the ones in S8A Fig. Empty lanes marked with “×”, and a repetitive DNA ladder lane also marked with “×” were not included in the final figure.

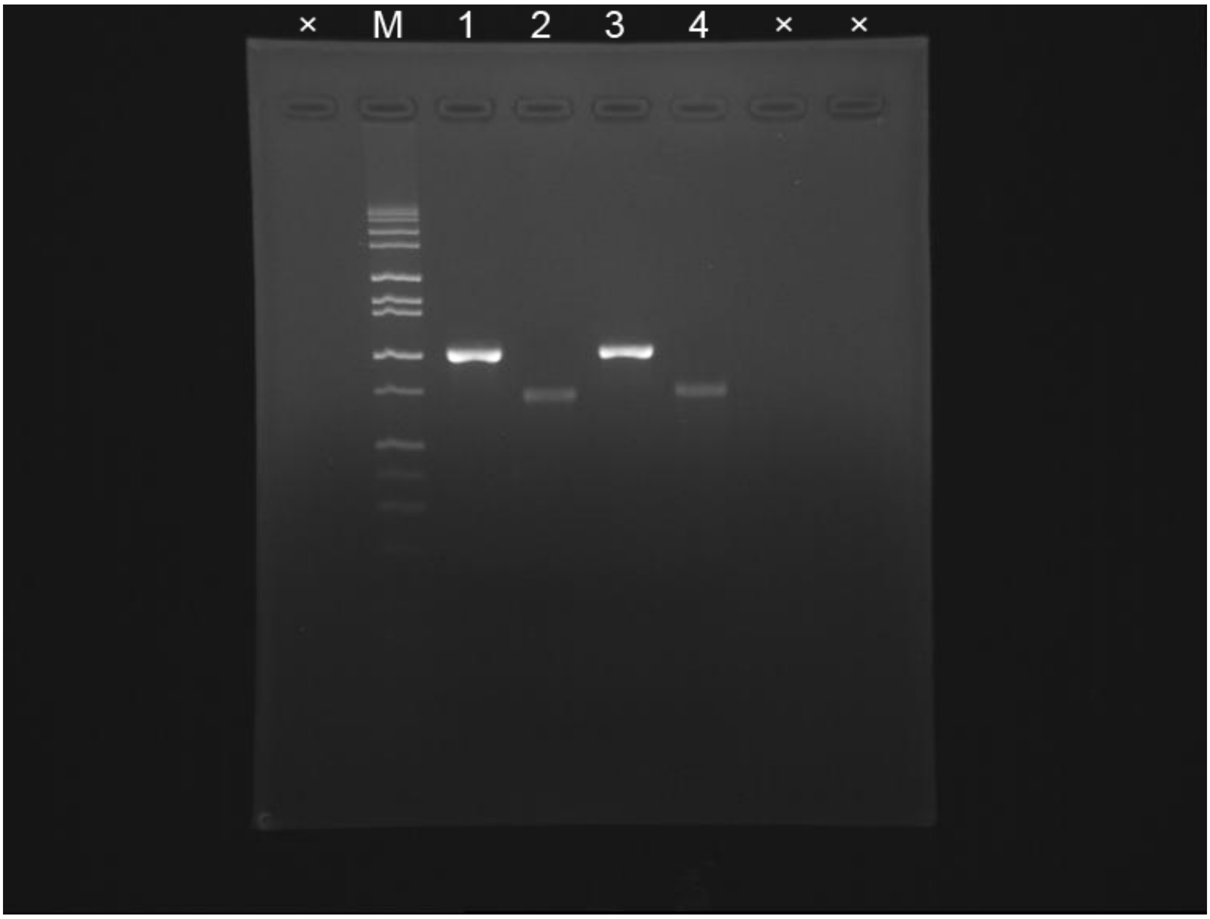

The above is the uncropped image for S8B Fig. The labels on each lane are the same as the ones in S8B Fig. Empty lanes marked with “×” were not included in the final figure.
